# American Academy of Dental Science

**Published:** 1901-03

**Authors:** 

**Affiliations:** American Academy of Dental Science


### AMERICAN ACADEMY OF DENTAL SCIENCE.

The regular monthly meeting of the American Academy of Dental Science was held at Young's Hotel, Boston, Wednesday evening, December 5, 1900, at six o'clock.

*President Pond.*—To-night we have a paper by Dr. W. E. Decker on "A Case of Orthodontia diagnosed and Progress watched by the X-Ray," a subject in which I do not doubt you will be deeply interested. I have the pleasure of presenting to you Dr. W. E. Decker.

(For Dr. Decker's paper, see page 141.)

DISCUSSION.

*Dr. Decker.*—I shall be pleased to answer questions on any points that have not been made perfectly clear.

*Dr. Clapp.*—Mr. President, I want to ask Dr. Decker if the pulp is still alive in the cuspid.

*Dr. Decker.*—So far as I know. The question has been asked me once or twice before, as it was brought down so rapidly. I watched it very carefully and often tested it. There has been no discoloration of the tooth, and I do not think that it is injured in any way. It was very much alive when I attempted to smooth the drill hole for a porcelain inlay.

*Dr. Clapp.*—The last time you tested it, was it all right?

*Dr. Decker.*—Yes.

*Dr. Baker.*—I think I can appreciate something of what has been done, and also the retaining of the teeth while he was waiting for the cuspid to come down, in such a way that nature would assist him. That is the vital point of his treatment. Any one who has done considerable regulating very well knows what nature will do to assist us, if we will only give her a chance. On the other hand, if he had retained these teeth in a fixed position, he would not have attained the results he has in the time it has taken to do this. I find that in retaining teeth it is a very important thing to give nature a chance to do its work to bring the teeth to good occlusion, and one will get much better results by doing so. I wish to thank Dr. Decker again for his valuable paper. In my opinion it is one of the best papers we have ever had before the society.

*Dr. Fillebrown.*—I observed the one specially new point that Dr. Decker has just alluded to, and I think it is well worth our remembering. That is, that he leaves those teeth on a hinge, so that nature can change the position of the roots after the teeth are put in place. Dr. Decker has demonstrated that nature will regulate a root if you only give it a chance after the crowns are put in place. That seems to me a decidedly original affair, and the most so of anything that we have had before this society in a long time. I compliment him.

*Dr. Andrews.*—I think, as has been said, that Dr. Decker's paper is one of the best we have had presented before us. I can appreciate parts of it. Some twenty years ago I brought before the Massachusetts Dental Society the case of a lady who said that she had never had a left superior permanent cuspid. I carefully examined her mouth, and made up my mind there was a cuspid in the jaw. I had at that time no X-ray to assist me. I made an opening in the jaw, and found that there was a cuspid tooth lying in a horizontal direction, and I opened to it and proceeded a good deal as the essayist did. I put a screw in and drew it down in place, and I must have turned the tooth, I should say, more than half an inch in the jaw tissue. I brought it down in place, and the tooth is there to-day, and I believe alive, strange as that statement may seem. It was certainly alive after I got it in place. I tested that when I filled the little cavity that I had made with the screw. The screw had worked loose, and I thought it best to fill its cavity. The tooth has grown in its new position solidly. The patient was not a young person. I should say she was twenty-four years of age when the operation was performed.

*Dr. Smith.*—It is such an easy matter to be misunderstood, that I rise to correct—or to be set right if I am wrong—the remarks that Dr. Fillebrown made in regard to the originality of the adjustment of this appliance so that the roots would move. This is not the first demonstration that the roots of teeth, as well as their crowns, can be moved. This has been amply demonstrated in recent publications. I sometimes think that the man of to-day who regulates teeth deserves quite as much credit in selecting the proper appliance from the many different devices as if he invented any new appliance himself.

I cannot see, however, but what we would get a similar effect, so far as tipping the points of the incisors in is concerned, if we used the regular old-fashioned vulcanite plate; you would certainly get the fulcrum. With the pins resting against the teeth there is a less amount of contact and a condition favorable for this oscillating movement.

This, Mr. President, is certainly a very interesting case. It has been handled in a most scientific way and has been presented in a most interesting manner, and shows the important strides that have been made in orthodontia.

Dr. Clapp is to show here, I believe, some negatives. One of the negatives is that of a case in which I am interested, and I thought the members might be interested to see the result in that case, and so I shall be pleased to show the models after Dr. Clapp has shown the negative.

*Dr. Fillebrown.*—My remarks applied to the method of retaining. Is not that a new thing?

*Dr. Decker.*—I have never seen that used, that I recall. I was casting about for some way of getting an arrangement upon all the teeth, yet which would not be rigid. I wanted tubes there because I wanted the teeth to swing. I did not wish to resort to Dr. Casey's method of moving the power up opposite the weight, because I did not want the patient remaining in the city so long when a fulcrum retainer and nature could be gradually doing the work for us. It seemed reasonable to me that if the arch would fall under the gentle pressure of the lips when teeth are extracted, this same pressure could be utilized in straightening teeth. The use of the tubes in this manner is certainly a very easy way of getting them in place. They were originally short solid tubes, each one opened by holding on the point of an instrument over a flame until red, then quickly pushed farther onto the tapering instrument, which opened it where it had been soldered. Then each was soldered with the back of the tube to the band, and each band set with the tube on the labial surface of the tooth. As the wire was slipped into the tubes on the posterior teeth, it fell right into the opened tubes on the anterior teeth. As I say, I do not recall ever seeing it used.

*Dr. Ainsworth.*—I want to add one word of appreciation and commendation of the paper. It is certainly an excellent result. One point I feel like emphasizing is the importance of doing this work slowly. We often hear of remarkable results being accomplished in a very short space of time; but I think it is a great deal better to go slowly and have the advantage of nature's assistance, which she freely gives when you work in harmony with her. If you have a tooth to elongate, it can be accomplished in a very few days, but in doing so you are very likely to draw it out of the process, and the process and gum are very slow to come down and cover the tooth, as they would if moved gradually.

I have found it a great advantage in cases where I am expecting nature to come to my assistance in a change of the process, to carry the case part way, and then leave it for some months, or through the summer vacation, and feel that I have accomplished results that could not be accomplished if the work was commenced and kept on continuously until completed.

This case shows a wonderful accomplishment, and suggests how much cases that we meet every day in the street might be improved if the public only appreciated the possibilities.

*Dr. Smith.*—I would like to ask Dr. Decker if he had any trouble in controlling the bleeding while setting the post into the tooth.

*Dr. Decker.*—Yes, I did. I went about that in a rather slow way, also. I did not set the post the day I did the lancing. After lancing, and chipping away the bone, I discovered my tooth, and put in a tent of cotton smeared with carbolized vaseline, to wedge the gum away, and I think it was two days later that I put in the post. Then it was a very easy matter to keep the tooth dry. The gums pushed away so that I could see it quite plainly.

*Dr. Smith.*—Did that post show?

*Dr. Decker.*—The hole is under the band at present.

*Dr. Clapp.*—I had the pleasure of watching this case from beginning to end, and I simply wish to emphasize the fact that this is the result of Dr. Decker's zest and patience and painstaking work.

*Dr. Smith.*—As the pictures that Dr. Clapp will show will probably lead to more discussion of Dr. Decker's paper, I move that we pass the subject.

Subject passed.

*President Pond.*—I have the pleasure of presenting Dr. Dwight M. Clapp.

*Dr. Clapp* (showing his negatives).—Here is a case of which Dr. Ainsworth can, perhaps, tell you something interesting.

*Dr. Ainsworth.*—I am sorry that I have not the models of this case here. It was a case of the right central standing next to the cuspid, and the right lateral immediately under the central. In place of the left central there were two supernumerary teeth. The left lateral was of good formation. The first question in my mind was which one of the supernumerary teeth we had best save and crown to match the other central. I did not believe, from the appearance of the case, that the other central was there; but Dr. Clapp's picture showed plainly that it was, but rather higher than the shadowgraph seems to indicate now. The two supernumeraries and the lateral which stood under the central were extracted. The belated central is now about half-way through the gums, and very much at an angle. We are just about adjusting an appliance to bring it down into place without drilling into it or in any way marring its beauty. It is a very interesting case, but the models show a clearer idea of it than I can give you. It is a case that demonstrates most beautifully the advantages of shadowgraphs in this work.

*Dr. Clapp.*—Here is a case of a deformed tooth. I think that this slide was made by Dr. Ainsworth, and he will be able to tell you why he made it, and the result of it. Will you tell them?

*Dr. Ainsworth.*—It was a cone-shaped lateral, the tooth perfectly sound, but an alveolar abscess had developed above it, and I queried whether the pulp was alive. Before photographing, I attempted to find the pulp cavity by drilling, but failed. By the aid of the X-ray, however, I was enabled to locate it, and got well up to the apex. Later I put on a crown, which much improved the appearance.

*Dr. Smith.*—This model, the negative of which Dr. Clapp has shown you, shows no trace of the missing central. The lateral came inside the arch, striking inside the lower central, and the first move was to bring the lateral out. There was more or less fulness, of course, outside. Pretty soon the cuspid tooth came through, and it was at that time that I had the patient go to Dr. Clapp for the X-ray picture.

This model, No. 2, shows the cuspid directly over the lateral, and the point of the right central incisor erupting at right angles to the left central incisor. The gum was pressed back and a gold cap made to fit the point. Strange to say, that cap stayed there perfectly until I got that tooth down and turned into place. Unfortunately, however, the tooth is an entirely different shaped tooth from its mate. It is more convex on one side and more concave on the other. The negative slightly shows that the tooth is deformed, but I did not think so much of it at the time. I was careful to tell the patient that in case this tooth proved to be a deformed tooth, it was wise to put it in place, because the pulp could be destroyed and a crown put on the root.

This model, No. 3, shows the tooth in place. The pulp of the tooth was alive at the last test. The lateral incisor, as you will see, is not in place. The next move is to bring this lateral out and move the root out, and give that fulness which will very much improve the looks of the mouth.

*Dr. Hopkins.*—The question of preserving the tooth alive during manipulation is spoken of as rather remarkable, but I think, when we remember the operation of nerve-stretching, we get a little light on this subject. You know frequently when the fingers become useless because of injury to the ulna nerve, the nerve is stretched, and during that stretching operation the surgeon does not hesitate to put on a pressure of forty or fifty pounds, and yet the nerve is uninjured and the patient is benefited. I think possibly the same thing happens in the more delicate nerve of the tooth, and that they will stand a good deal more stretching than we have ever thought it possible to apply without injury.

*Dr. Fillebrown.*—There is no tissue that is produced faster than nerve-tissue. You can sever the nerve and leave the ends a considerable distance apart. The space will fill up directly and the nerve perform all the functions of which it was capable before it was injured. So if you put a nerve on the stretch and keep it extended, it will elongate and that very rapidly. I think the trouble which so often obtains where you destroy the pulp by moving the tooth will be found in cases where the tooth is tipped and the nerve pinched. If you pinch it, you will produce strangulation, and of course a dead pulp. But so long as you keep the apex of the root free and clear from any pinching, and simply stretch it by moving the tooth, the nerve-tissue will be produced quite as fast as any of the other tissues. I think that is the true explanation of it, and why we can now do so much without menace to the life of the pulp.

*Dr. H. A. Baker.*—I did not fully appreciate the advantages of the X-ray in dentistry until quite recently. Something like six months ago a dentist brought his daughter, also models, to me for my opinion in a case of orthodontia. In place of a left lower second bicuspid was a temporary molar, and it was impossible to tell whether the bicuspid was beneath the molar or not. I advised him to go to Dr. Clapp and subject it to the X-ray, which he did, and yesterday he sent me a photograph of it which shows no indication of an approaching bicuspid. Had we extracted the temporary molar on the supposition that the bicuspid was beneath, it would have been bad practice. Now that the X-ray has shown there is no bicuspid, our way is clear, for the molar is perfectly sound and solid in its place, and in my opinion we will obtain much better results by letting it remain than to have no tooth there at all.

Subject passed.

INCIDENTS OF PRACTICE.

*Dr. Brackett.*—I had a little incident of practice this afternoon that pleased me. A young lady came from school at two o'clock to keep an appointment of an hour and a half. We had a storm of sleet, and she came without rubbers, with damp feet, and sat down in my chair in a very uncomfortable and unsafe condition. Seeing her uncomfortable, I was uncomfortable myself, and I turned over in my mind several plans for trying to help her. Finally, I remembered that I had in the house an electric foot-warmer that for its operation simply needs connection with any lamp-socket. I made a connection, put the little flat stove on the foot-rest of the chair, and very soon her feet were getting dry, and she was warm and comfortable. The cost of this electric heater was about six dollars. They may be procured of any dealer in electrical apparatus, and are convenient and serviceable wherever electricity is installed.

*Dr. Andrews.*—I have one of these little copper gas-stoves, and just at the foot of my chair I place it in cold days, to warm the feet. I find it very comfortable and very fine. It not only warms the patient's feet, but it dries the clothing.

On motion of Dr. Smith, a vote of thanks was extended to Dr. Decker for his very scientific paper.

CHARLES H. TAFT, D.M.D.,
*Editor American Academy of Dental Science.*

